# Ultrasonic-assisted enzymatic extraction of sulfated polysaccharide from Skipjack tuna by-products

**DOI:** 10.1016/j.ultsonch.2023.106385

**Published:** 2023-03-27

**Authors:** Shahab Naghdi, Masoud Rezaei, Mehdi Tabarsa, Mehdi Abdollahi

**Affiliations:** a Seafood Processing Department, Marine Sciences Faculty, Tarbiat Modares University, Noor, Iran; b Department of Life Sciences–Food and Nutrition Science, Chalmers University of Technology, SE 412 96 Gothenburg, Sweden

**Keywords:** *Katsuwonus pelamis*, Sulfated polysaccharide, Ultrasonic-assisted enzymatic extraction, Functional activities, Biological properties

## Abstract

The effect of ultrasound pretreatment on extraction efficiency of sulfate polysaccharides (SPs) using alcalase from different by-products of Skipjack tuna including head, bone and skin was evaluated. Structural, functional, antioxidant and antibacterial properties of the recovered SPs using the ultrasound-enzyme and enzymatic method were also investigated. Ultrasound pretreatment significantly increased the extraction yield of SPs from all the three by-products compared with the conventional enzymatic method. All extracted SPs showed high antioxidant potential in terms of ABTS, DPPH and ferrous chelating activities where the ultrasound treatment enhanced antioxidant activities of the SPs. The SPs exerted strong inhibiting activity against various Gram-positive and Gram-negative bacteria. The ultrasound treatment remarkably increased antibacterial activity of the SPs against *L. monocytogenes* but its effect on other bacteria was dependent on the source of the SPs. Altogether, the results suggest that ultrasound pretreatment during enzymatic extraction of SPs from tuna by-products can be a promising approach to improve extraction yield but also bioactivity of the extracted polysaccharides.

## Graphical abstract

**Figure:**
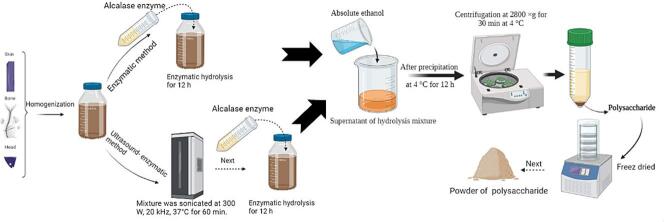

## 1 Introduction

In the current decade, consumer’s desires have greatly changed to use food containing the least amounts of chemical additives to improve their health and lifestyle [1]. As a result, people tend to use dietary supplements containing bioactive compounds such as sulfated polysaccharides, proteins, polyunsaturated fatty acids (PUFAs), pigments and vitamins isolated from various natural sources including plants [2], animals [3] and microbial sources [4]. Currently, SPs are usually obtained from pig (skins, hides, and bones) in industrial scales [5]. However, there are some problems with using pig as a source of SPs such as the transmission possibility of influenza from pig and religious limitation in some countries [5], [6]. Therefore, researcher’s attention has turned to marine sources such as fish by-products [5], [7]. For example, tuna fish accounts for about 4.6% of total global captured fish annually which creates a huge volume of by-products including head, viscera, and tail after processing [8] that usually goes to fish meal production. These by-products can be however a great candidate to extract SPs [9], [10].

Till now, different conventional extraction protocols including solvent [11], supercritical [12], microwave-assisted [13] and ultrasound-assisted [14] methods have been employed to isolate bioactive compounds. Additionally, non-conventional methods which are known as green methods that are eco-friendly, and non-toxic have been recently adopted by researchers. More innovative methods can be created by combining different conventional and non-conventional methods such as enzyme-ultrasound [15], ultrasonic-microwave [16], microwave- enzyme [17] assisted methods. This can also provide an opportunity to optimize quality of SPs since extraction method can play a significant role in defining different properties of extracted SPs [18], [19].

Enzymatic extraction methods have been typically used to recover sulfated polysaccharides from marine resources [9], [20]. In addition to reducing economic viability, enzyme treatment has also yielded low extraction rates [18]. Applying ultrasonication can be a promising approach to increase extraction yield and decrease extraction time by destruction of the biomass before enzyme treatment [18]. When the ultrasound process is used the cavitation phenomenon can destroy cell walls, inducing cell content leakage. This facilitate the access of the enzyme and the target compounds or its substrate and improve extraction yield [19]. Previously, Alboofetileh et al. [21], who used three extraction methods including enzyme, ultrasound, and enzyme-ultrasound to extract SPs from *Nizamuddinia zanardinii*, reported that enzyme-ultrasonic isolated fucoidan showed the highest extraction yield (7.87%) while ultrasound by alone resulted in the lowest yield (3.6%). Also, Liao et al. [15] studied the extraction of polysaccharides from *Corbicula fluminea* by ultrasonic-assisted enzymatic (UAEE) method. They found that the yield of UAEE in 32 min was higher than that of EAE in 4 h. However, to the best of our knowledge till now, the use of the enzyme-ultrasonic extraction method to extract SPs from fish and fish by-products have been neglected. Therefore, the present study was aimed to compare the efficiency of an enzymatic method and its combination with ultrasonic in extraction of SPs from the skin, bones, and head of Skipjack tuna (*Katsuwonus pelamis*). The effect of the two extraction methods on physicochemical antimicrobial and antioxidant properties of the recovered SPs were also evaluated.

## 2 Material and methods

### 2.1 Sample collection

Skipjack tuna by-products (skin, bones, and head) were purchased from a tuna processing company in the Babolsar region of Iran. All parts of fish by-products were packaged in polyethylene bags and transferred to the laboratory while they were covered by ice at the ratio of 1:3 fish by-product to ice approximately. As soon arriving to the lab, the by-products were cleaned with tap water to remove pigments and packed in a polyethylene bags and saved at −40 °C until used for extraction of SPs [5].

### 2.2 Extraction of sulfated polysaccharide

#### 2.2.1 Conventional enzymatic extraction

The extraction of sulfated polysaccharides from fish by-products carried out by ethanol precipitation process described by Jridi et al. [5] with some modifications. In brief, 100 g of ground by-product was suspended in 100 mL of distilled water. For inactivation of endogenous enzymes, the mixture was heated at 90 °C for 15 min and kept until its temperature reached to room temperature. Then, the pH of the mixture was adjusted to 7.5 and Alcalase® was added to the cooled mixture at a level of 500 U/g samples and enzymatic proteolysis was continued for 12 h at 50 °C. Then, the mixture was centrifuged at 2800 × g for 30 min at 4 °C and the supernatant was gathered. The supernatant was precipitated by absolute ethanol (w/2v) at 4 °C for 12 h and centrifuged at 2800 × g for 30 min at 4 °C. Finally, the pellets were redissolved in distilled water and lyophilized to obtain the SPs. The SPs recovered from Skipjack tuna skin, bones, and head by the enzymatic method named skin-Esp, bone-Esp, and head-Esp, respectively and those extracted with ultrasound-enzyme method named skin-UEsp, bone-UEsp, and head-UEsp, respectively.

#### 2.2.2 Ultrasonic-assisted enzymatic extraction

As before, 100 g of each minced by-product was mixed with 100 mL of water and treated for 60 min by a 20 kHz ultrasound (UHP-400, Topsonic, Iran) under 300 W at 37 °C. The length and diameter of the probe were 14 and 1.3 cm, respectively, of which 5 cm was placed inside the mixture. The time of the on/off pulses was 6 sec/2 sec. The mixture was stirred during the process and use ice to keep temperature constant.

### 2.3 Chemical composition

The phenol–sulfuric acid method at 490 nm with D-fucose as the standard used to determine the total carbohydrate content [22]. To measure the content of protein in different extracts, Lowry method with bovine serum albumin as the standard was used [23]. The BaCl_2_ gelatine method at 360 nm was used to determine the content of total sulfate [24]. The m-hydroxybiphenyl method at 525 nm with D-glucuronic acid as the standard was applied to measure uronic acid content [25].

### 2.4 Monosaccharide composition of samples

The monosaccharide composition of different polysaccharides was determined using the method reported by Alboofetileh et al. [21]. Briefly, 2 M trifluoroacetic acid (TFA) was added to the samples and incubated for 2 h at 121 °C to hydrolyse the SPs. Then, TFA evaporated by a dried stream of nitrogen at 50 °C. The sodium borohydride and acetic anhydride were used to reduce and acetylate the hydrolysed samples. The final products were analysed by gas chromatography-mass spectrometry (GC–MS). Glucuronic acid, galacturonic acid, mannose, rhamnose and xylose were applied as a monosaccharide standard. The results were reported as the relative area of the peaks.

### 2.5 Molecular weight distribution

A HPSEC–UV–MALLS–RI system (high-performance size exclusion chromatography column coupled to UV, multi-angle laser light scattering, and refractive index detection) was used to determine the molecular weight of the extracted SPs. The sample preparation and determination of the average molecular weight (Mw) of sulfated polysaccharides was performed as previously reported by Alboofetileh et al. [26].

### 2.6 FT-IR spectroscopy

After mixing SPs with KBr, the powdered mixtures were loaded into the testing cell. The spectra of the samples were read at 400–4000 cm^−1^ region using a Fourier transform IR spectrophotometer (Bruker Instruments, Billerica, USA) to detect functional groups [5].

### 2.7 Differential scanning calorimetry (DSC) analysis

The method developed by Trigui et al. [27] was used to assess the glass transformation temperatures of the SPs. Herein, 5 mg of each polysaccharide sample was settled in a standard aluminium lid and heated at a temperature range of 25–200 °C at a rate of 5 °C/min under a nitrogen protection atmosphere.

### 2.8 X-ray diffraction

The method described by Trigui et al. [27] were applied to determine the X-ray diffraction (XRD) pattern of crude polysaccharides with 2θ ranging from 2°–80° at room temperature using a X-ray diffractometer (Siemens D 5000, Bruker, Germany). The step size and time per step were 0.02° and 5 sec/step, respectively.

## 3.1 Functional properties of extracted SPs

### 3.1.1 Foaming properties of SPs

The foaming properties of sulfated polysaccharides were evaluated following the reported protocol by Yuan et al. [28]. First, 5 mL of each SPs solution at a concentration of 1% w/v was homogenized for 3 min at 2000 rpm at room temperature and the height of the generated foam was measured and its foaming capacity (FC) was calculated using equation (1). To determine foam stability (FS), the whipped solution was left for 30 min at room temperature and its height was measured again and FS was calculated using equation (2).

(1) $$FC\left(\%\right)=\;\left(\mathrm{VT}-V0/V0\right)\;\times \;100$$

(2) $$FS\left(\%\right)=\;\left(\mathrm{Vt}-V0/V0\right)\;\times \;100$$

Here, V_T_ is the total volume after whipping; V_0_ is the volume before whipping and Vt is the total volume after leaving at room temperature for 30 min.

### 3.1.2 Emulsifying activities of SPs

Emulsifying properties of SPs were assessed in the presence of sunflower oil and soybean oil [29]. Briefly, a 1 % (w/v) solution of SPs combined with sunflower oil and soybean oil, and vibrantly vortexed for 2 min. Then, the mixture was left for 24 h and emulsification index (E24) calculated using equation (3).

(3) $$E_{24}=\;\left(H_{e}/H_{t}\right)\;\times \;100$$

Here, H_e_ and H_t_ are the height of the emulsion layer and the total height of the mixture, respectively.

## 3.2 Determination of antioxidant activities of SPs

### 3.2.1 DPPH scavenging activity of SPs

DPPH scavenging activity was measured using the method described by Alboofetileh et al. [30]. In summary, approximately 100 μL of each sample solution with concentration of 0.5, 1, 1.5, and 2 mg/mL was transferred into 96-well microplates and 100 μL of DPPH solution was added to each well. Then, the mixture was incubated for 30 min in the dark place at room temperature. Finally, the absorbance of the solution was read by an ELISA microplate reader at 515 nm. Ascorbic acid (100 μg/mL) was used as a positive control. The following equation was used for calculating the DPPH radical scavenging activity:

(4) $$\mathrm{DPPH}\;scavenging\;activity\;\%=\;\left[\;Ac\;--\;As/Ac\right]\;\times \;100$$

where Ac is the absorbance of the control (100 μL of ethanol with 100 μL of the DPPH solution) and as the absorbance of sulfated polysaccharides sample solution.

### 3.2.2 ABTS scavenging activity of SPs

ABTS scavenging activity of the SPs was tested according the method of Saravana et al. [29] with slight modifications. Briefly, SPs were prepared in serial dilutions including several concentrations (0.5, 1, 1.5 and 2 mg/mL). Then, ABTS radical cation was prepared by mixing 7 mM ABTS in 2.45 mM potassium persulfate and incubated for 16 h in a dark place at room temperature. After that, the ABTS solution was diluted by ethanol until the absorbance of the ABTS solution reached 0.7 at 734 nm. The ABTS scavenging activity of the different dilutions of SP was determined by transferring 50 μL of the prepared samples into 96-well microplates. After that, 150 μL of ABTS solution was added to each well and incubated at darkness for 20 min. Finally, an ELISA microplate reader was used to measure the absorbance of samples at 734 nm. Ascorbic acid (100 μg/mL) was used as a positive control. The ABTS radical scavenging activity was calculated using the following equation:

(5) $$\mathrm{ABTS}\;scavenging\;activity\;\%=\;\left[\mathrm{Ac}--As/Ac\right]\;\times 100$$

where Ac was the absorbance of the control (50 μL of ethanol with 150 μL of the ABTS solution), and As was the absorbance of SP solutions. The experiments were carried out in triplicate.

### 3.2.3 Ferric reducing power of SPs

This test was carried out according the method described by Jridi et al. [9]. 100 μL of each SP solutions at different concentrations of 0.5, 1, 1.5, and 2 mg/mL was added to 50 μL of 2 mM FeCl_2_ and 450 μL of distilled water. The mixtures were incubated at room temperature for 5 min and 200 μL of 5 mM ferrozine solution was added to start the reaction. After shaking the mixtures, they were incubated at room temperature for 10 min. EDTA was used as a positive control. Finally, the absorbance of different solutions was measured at 562 nm, and the chelating activity (%) was calculated using equation (6):

(6) $$\mathrm{Ferric}\;reducing\;power\left(\%\right)=\left[\left(\mathrm{ODC}+ODB-ODS\right)/ODC\right]\times 100$$

where ODC, ODB and ODS represent the absorbance of the control, the blank and the sample reaction tubes, respectively. The experiments were carried out in triplicate.

## 3.3 Antibacterial activity of SPs

### 3.3.1 Bacterial strains

The stocks of two Gram-positive bacteria (*Staphylococcus aureus* and *Listeria monocytogenes*) and two Gram-negative bacteria (*Escherichia coli* and *Salmonella enterica*) were purchased from Pastor institute of Iran. Each bacterial strain was mixed with tryptic soy broth (TSB) supplemented with 30% glycerol and kept at −20 °C until use. Before inoculation of the bacteria, all the used bacteria were cultured in 10 mL TSB at 37 °C for 24 h. Then, the culture medium was centrifuged at 3400 rpm for 10 min to separate the grown bacteria from the medium. Next, they were washed with 0.85% NaCl solution and centrifuged twice for 15 min at 3400 × *g*. Finally, to adjust the concentration of bacteria to1 × 10^8^ CFU/mL, we used NaCl (0.85%) suspension as diluter until the optical density (OD) of a bacterial suspension at 600 nm reached 0.1. The suspension was then diluted to provide a cell concentration of 1 × 10^5^ CFU/mL.

### 3.3.2 Agar diffusion method

Antibacterial activity of SPs was evaluated against the Gram-positive (*Staphylococcus aureus* and *Listeria monocytogenes*) and Gram-negative bacteria (*Escherichia coli* and *Salmonella enterica*) as described by Jiang et al. [2]. All bacterial strains (a density of 1 × 10^5^ CFU/mL) were uniformly swabbed on the surface of the Tryptic Soy Agar medium. Then, punched discs, which were sterilized at 121 °C for 30 min, soaked by 20 µL of different concentrations (1 and 2 mg/mL) of SPs. After that, the discs were put on the surface of the plates. The plates were incubated at 37 °C for 24 h and the inhibition zone was measured (data expressed as mm). All the experiments were carried out in triplicate.

## 3.4 Statistical analysis

One-way ANOVA and Duncan's test (p < 0.05) were used to determine the differences in various evaluated tests of SPs.

## 4 Results and discussion

### 4.1 Effect of extraction methods on the yield of crude SPs

The extraction yields and composition of SPs obtained by ultrasound-enzyme or enzyme extraction methods from skin, bones, and head of Skipjack tuna are shown in Table 1. The obtained amounts of SPs by the enzyme-assisted extraction method from head, skin, and bone were 2.42%, 3.1%, and 2.61% (based on wet weights), respectively, while extraction yield for those extracted by ultrasound-enzyme extraction method were 3.01%, 3.57%, and 2.93%, respectively (p < 0.05). It is generally known that the origin of raw material is the main factor defining extraction yield of SPs varying between 0.1% and 8.9% among different biomasses [31]. Besides, it is well known that the polysaccharide extraction yield is greatly influenced by the extraction methods [19], [32]. As shown in Table 1, the ultrasound-enzyme extraction method was more effective than the enzyme method in extraction of SPs regardless of the used by-product. This can be explained by the fact that when the mixture is sonicated, the biomass is disintegrated by the cavitation which facilitates the activity of the enzyme [19], [33]. The extraction yield of SPs from Bullet tuna (*Auxis Rochei*) by-products were 1.3%, 0.93%, and 3.74% for head, skin, and bone, respectively as reported by Jridi et al. [5] which showed slight difference with our results. Further, extraction yield of SPs from the skin of *Mustelus mustelus* by ethanol precipitation was also 2.9% [7]. Interestingly, a similar result was obtained by Alboofetileh et al. [21] who extracted SPs from *Nizamuddinia zanardinii* by enzyme, ultrasound, and enzyme-ultrasound methods, where the highest yield SPs was obtained using the enzyme-ultrasound method with 7.87%. Based on the present results, it can be concluded that application of ultrasound during enzymatic extraction of SPs can promote extraction efficiency of SPs from fish by-products.

**Table 1:** Chemical and monosaccharide compositions of polysaccharides extracted from Skipjack tuna by-products (skin, bones and head) using ultrasound-enzyme or enzyme extraction method.

Bullet tuna by-product parts	Skin		Bone		Head	
	Ultrasound-Enzyme	Enzyme	Ultrasound-Enzyme	Enzyme	Ultrasound-Enzyme	Enzyme
Yields (%)	3.57 ± 0.43 ^a^	3.10 ± 0.22 ^ab^	2.93 ± 0.14^b^	2.61 ± 0.16 ^bc^	3.01 ± 0.13^b^	2.42 ± 0.37^c^
Total sugars (%)	53.66 ± 1.16 ^ab^	52.44 ± 0.63^b^	55.16 ± 1.75 ^a^	55.44 ± 1.41 ^a^	51.77 ± 1.25^b^	49.11 ± 1.57^c^
Proteins (%)	19.45 ± 1.44 ^a^	17.65 ± 1.19 ^a^	18.14 ± 0.98 ^a^	17.86 ± 0.80 ^a^	15.17 ± 0.56^b^	15.34 ± 1.6^b^
Uronic acid (%)	8.19 ± 0.24 ^a^	7.14 ± 0.67^b^	7.96 ± 0.16 ^ab^	7.12 ± 0.22^b^	5.06 ± 0.49^c^	4.20 ± 0.62^c^
Sulfates (%)	8.02 ± 0.16 ^d^	6.80 ± 0.41^d^	19.93 ± 0.49 ^a^	18.41 ± 0.78^b^	18.16 ± 0.53^b^	14.23 ± 1.27^c^
Monosaccharides						
Rhamnose (%)	15.9	16.4	15.3	16.3	13.4	12.3
Xylose (%)	16.6	17.3	17.1	16.2	31.6	33.8
Mannose (%)	17.3	18.7	16.8	17.3	24.3	23.7
GlcA (%)	24.5	23.2	25.9	24.5	25.8	24.9
GalA (%)	25.7	24.4	24.9	25.7	4.9	5.6

GlcA (glucuronic acid) and GalA (galacturonic acid). Data are calculated based on wet weights. a,b Different letters in the same raw indicate significant differences (p < 0.05). ⁎ % of dry weight.

### 4.2 Chemical and molecular characterization of SPs

Protein contents of SPs were found to be 19.45%, 17.65%, 18.14%, 17.86%, 15.17%, and 15.34% for skin-UEsp, skin-Esp, bone-UEsp, bone-Esp, head-UEsp, and head-Esp, respectively. In most cases, ultrasound pre-treatment increased the protein content of SPs. However, previous studies have reported that the protein content of SPs obtained from fish by-products is very diverse which could be related to the nature of the source biomass used for extraction of SPs [9], [15]. Uronic acid contents of SPs are shown in Table 1. SPs extracted by the Ultrasound-Enzyme method showed higher uronic acid content than those obtained by the enzyme method (p < 0.05). The highest content of uronic acid was found in skin-UEsp, while the lowest one was in head-Esp which was in line with the results obtained in previous studies [5], [9]. The sulfate contents of SPs were varying significantly from 8.02% to 19.93 % (p < 0.05), and bone-UEsp and skin-UEsp showed the highest and the lowest values, respectively. The nature of the material and type of extraction are known as key factors affecting on the sulfate content of SPs [20], [34]. Similarly, Jridi et al. [5] reported the highest sulfate content in SPs from head while the lowest content was related SPs from the skin of Bullet tuna. A number studies have stated that the increase of sulfate content in sulfated polysaccharides could enhance its bioactivity [34], [35].

### 4.3 Monosaccharide composition

The monosaccharide composition of different SPs are displayed in Table 1. All samples showed a mixed composition, with high content of xylose, mannose, and glucuronic acid (GlcA), showing some differences in the content of monosaccharides among the SPS. Ultrasound pretreatment could increase the content of rhamnose (in the obtained sulfated polysaccharide of skine and bone), xylose (in the obtained sulfated polysaccharide of skine and head), mannose (in the obtained sulfated polysaccharide of skine and bone), glucuronic acid (in the obtained sulfated polysaccharide of skine and bone), and galacturonic acid (in the obtained sulfated polysaccharide of bone and head). The present results are in line with those reported by Jridi et al. [5], who studied the effect of cetylpyridinium chloride (CPC) or ethanol precipitation on different features of polysaccharides extracted from Bullet tuna (*Auxis Rochei*) by-products, and documented that the bioactivity of sulfated polysaccharides depends on the monosaccharide contents in glycosaminoglycans (GAGs). All used standard monosaccharides in the present work were also seen in the study conducted by Abdelhedi et al. [7], who also showed CPC precipitation was more effective in precipitating GlcA and GalA from smooth-hound viscera than ethanol. Moreover, it has been previously reported that the content of monosaccharides in the SPs from different parts of fish or marine animal is affected by the organ of origin or the extraction method [5], [7]. As reported by Chen et al. [36], ultrasonic treatment of polysaccharides during extraction caused changes in the molecular ratio of monosaccharides.

### 4.4 Molecular weight of SPs

**Fig. 1:**
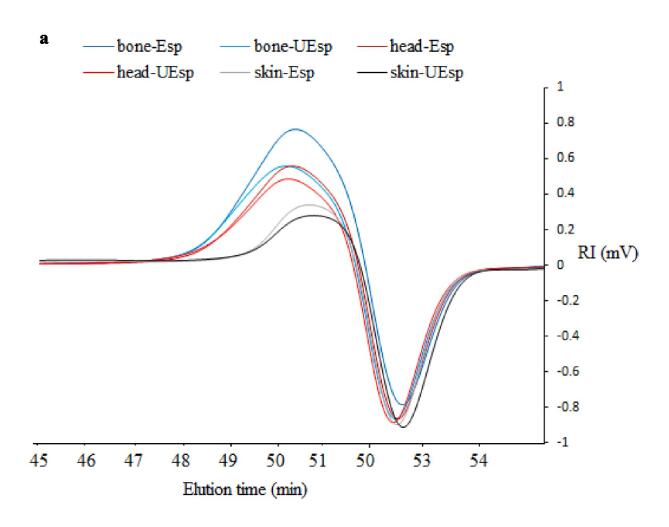

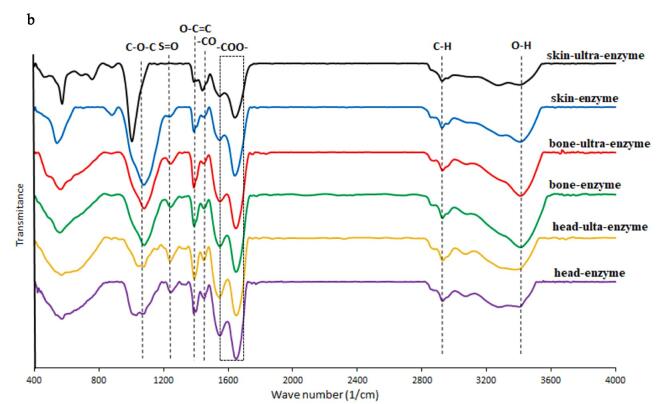
**A** RI chromatograms of extracted sulfated polysaccharides from Skipjack tuna by-products (skin, bones and head) using ultrasound-enzyme or enzyme extraction method. bone-Esp: the sulfated polysaccharide extracted by enzyme-assisted extraction method from bone; bone-UEsp, the sulfated polysaccharide extracted by ultrasound-enzymatic extraction method from bone, head-Esp: the sulfated polysaccharide extracted by enzyme-assisted extraction method from head, head-UEsp: the sulfated polysaccharide extracted by ultrasound-enzymatic extraction method from head, skin-Esp: the sulfated polysaccharide extracted by enzyme-assisted extraction method from skin, skin-UEsp: The sulfated polysaccharide extracted by ultrasound-enzymatic extraction method from skin. **1b.** FT-IR Spectra of extracted sulfated polysaccharides from Skipjack tuna by-products (skin, bones and head) using ultrasound-enzyme or enzyme extraction method.

Fig. 1a shows the superimposed RI chromatograms for the extracted SPs. All the SPs had one main peak at the elution time of 50 min which its intensity was different among the SPs. The molecular weight of SPs from bone-Esp, bone-UEsp, head-Esp, head-UEsp, skin-Esp, and skin-UEsp was 46.25 kDa, 45.65 kDa, 35.8 kDa, 33.5 kDa, 20.6 kDa, and 19.1 kDa, respectively. As can been seen, type of by-product has a big impact on the molecular wright of extracted SPs where bone resulted in SPs with the highest molecular weight followed by head and skin. Ultrasound treatment also resulted in SPs with slightly lower molecular weight compared with SPs recovered with the enzyme treatment by alone from each by-product. The molecular weight of all extracted SPs was lower than those reported by Jridi et al. [5], who extracted SPs from Bullet tuna (*Auxis Rochei*) by-products by enzymatic method. Souissi et al. [35] extracted SPs from razor clam, Solen marginatus, and reported two major peaks at 1075 kDa and 237.9 kDa for the recovered SPs. It has been reported that the molecular weight of polysaccharides can be dependent on the extraction process, purification techniques, and deproteinization treatment [21], [35]. As compared to enzymatic extraction based on Liao et al. [15] ultrasound-enzymatic extraction significantly reduced the average molecular weight of polysaccharides extracted from *Corbicula fluminea*. A study by Cheung et al. [37] revealed that ultrasound extraction of polysaccharide–protein complexes from *Grifola frondosa*, *Coriolus versicolor*, and *Lentinus edodes* increased the peak area and the number of lower-molecular weight peaks or shifted the molecular weight distribution from high to low molecular weight compared to a common extraction method, which was in parallel with our results. Additionally, Li et al. [38] reported that polysaccharides' molecular weight decreased after overheating and overtreatment with ultrasonic energy.

### 4.5 FT-IR spectroscopy

The structure of extracted sulfated polysaccharides and their functional groups are distinguished via FT-IR spectroscopy at 4000–400 cm^−1^ region (Fig. 1 b). As shown in the FT-IR spectra of SPs, there was a typical wide-stretching peak related to a hydroxyl group of carbohydrates at 3400 cm^−1^. The weak peak around 2900 cm^−1^ were related to the C–H stretching and bending vibrations representing the fucose methyl group [34], [39]. The presence of bonds related to the asymmetric stretching vibration of –COO- was confirmed by a peak around 1600 cm^−1^, and also the C-O-C bond was related to the presence of uronic acid [6], [9], [39]. Moreover, the stretching vibrations around 1200 cm^−1^ and 584 cm^−1^ are attributed to the S-O sulfate group [6], [9], [40]. In addition, the signals observed at around 1030 cm^−1^ are assigned to the symmetric of an ether sulfate group (RO–SO ^-3^) [35]. Besides, C-O bond was observed at 1450 cm^−1^ [9]. Based on the obtained results from the infrared spectra of SPs, it was clear that all extracts showed a little difference in infrared spectra but there was no big impact from ultrasound treatment on the structure of extracted SPs [31]. This result was in agreement with the results reported by Olawuyi et al. [19] who extracted polysaccharides from okra by enzyme-assisted, combined enzyme-ultrasonic methods, hot-water, and ultrasonic methods. They found that the same peak shape was observed in all extracted polysaccharides, indicating that extraction methods had no effect on structural conformation.

### 4.6 Differential scanning calorimetry (DSC) analysis

**Fig. 2:**
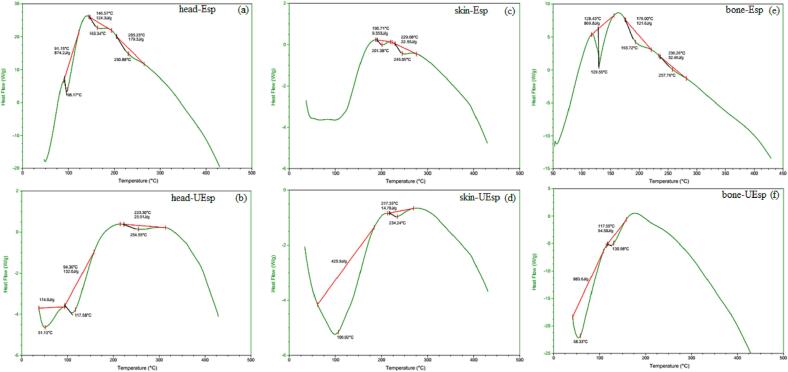
DSC thermographs of extracted sulfated polysaccharides from Skipjack tuna by-products including (a) head-Esp, (b) head-UEsp, (c) skin-Esp, (d) skin-UEsp, (e) bone-Esp and (f) bone-UEsp. bone-Esp: the sulfated polysaccharide extracted by enzyme-assisted extraction method from bone; bone-UEsp, the sulfated polysaccharide extracted by ultrasound-enzymatic extraction method from bone, head-Esp: the sulfated polysaccharide extracted by enzyme-assisted extraction method from head, head-UEsp: the sulfated polysaccharide extracted by ultrasound-enzymatic extraction method from head, skin-Esp: the sulfated polysaccharide extracted by enzyme-assisted extraction method from skin, skin-UEsp: The sulfated polysaccharide extracted by ultrasound-enzymatic extraction method from skin.

Fig. 2, shows DSC results for polysaccharides obtained from different tuna by-products using the two extraction methods. As can be seen, extraction method affected on thermal properties of SPs resulting in different thermographs in SPs extracted from similar by-products but using different extraction methods. At the beginning of heating process, a decrease at heat flow of the SPs at 100 °C occurred which could be due to the evaporation of water in the samples. Also, a different enthalpy was observed for each sample which should be related to differences in chemical structure of the samples and their hydrophilic groups [35], [41]. With further increase in temperature, another peak appeared for all samples at around 200 °C which should be related to the melting temperature of the samples. The changes that are observed in the DSC graphs of extracted SPs are due to variances in the moisture content and different structures of the polysaccharides, which have been caused by the extraction process [41], [42]. According to the DSC curves, apparent ΔH indicates the energy required to disrupt hydrogen bonds within junction zones [43]. For example, higher ΔH can be attributed to stronger carbohydrate–water interactions and better microstructures [43]. All the SPs recovered with the aid of ultrasound pretreatment showed lower ΔH compared with their counterparts recovered without ultrasournd. The lower ΔH of the untrasound treated samples may be due reduction of structural stability of the SPs resulted in a lower thermal stability [43]. This is also in line with the lower molecular weight measured in the SPs produced with the aid of ultrasound (see Fig. 1a).

### 4.7 X-ray diffraction

**Fig. 3:**
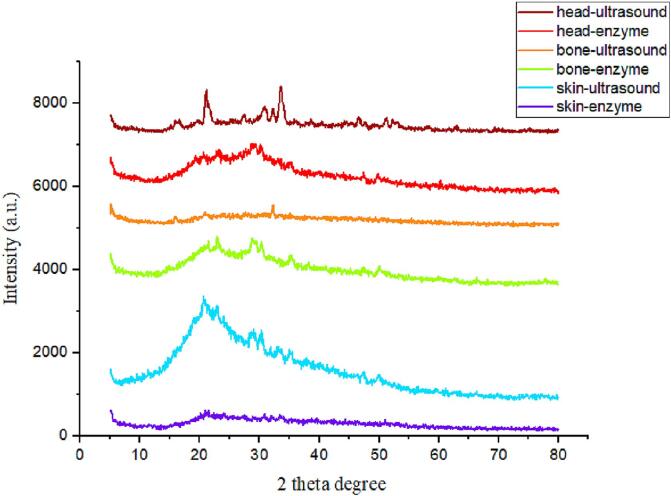
XRD graph of extracted sulfated polysaccharides from Skipjack tuna by-products (skin, bones and head) using ultrasound-enzyme or enzyme extraction method.

The XRD technique is a powerful tool for unraveling polysaccharide structures. In this method, crystalline structures of materials are analyzed [2]. As depicted in the XRD graph, there is a peak at 20°which is sharper and more clear in some of the SPs but it appeared wider in some other SPs (Fig. 3). Fig. 3 indicates that all SPs were amorphous polymers because their peak regions were around angle 20 [2]. Additionally, they are semicrystalline polymers with low crystallinity [29]. Some differences can be seen in the in the intensity of the peak at 20°but it was very much dependent on the source of extraction resulting in difference effect from ultrasound too. This makes drawing a conclution about the effect of ultrasound of crystalin structure of the SPs difficult since the peak intensity increased in the SPs from skin with ultrasound treatment while it decreased in the SPs from head and bone with ultrasound. Consequently, these structural arrangements directly affect various properties of SPs such as tensile strength, flexibility, solubility, swelling and dispersibility [12], [29].

### 4.8 Functional properties of extracted SPs

#### 4.8.1 Foaming capacity and foam stability

**Fig. 4:**
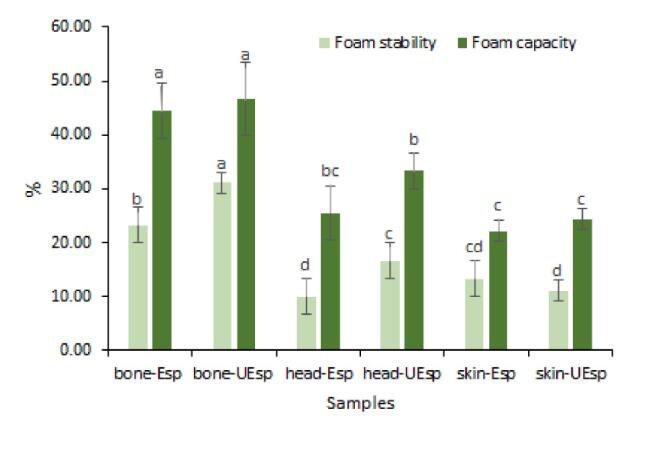
Foaming properties of extracted sulfated polysaccharides from Skipjack tuna by-products (skin, bones and head) using ultrasound-enzyme or enzyme extraction method. The results were expressed as mean value ± SD (n = 3). Different letters within the same figure mean statistical difference (p < 0.05). bone-Esp: the sulfated polysaccharide extracted by enzyme-assisted extraction method from bone; bone-UEsp, the sulfated polysaccharide extracted by ultrasound-enzymatic extraction method from bone, head-Esp: the sulfated polysaccharide extracted by enzyme-assisted extraction method from head, head-UEsp: the sulfated polysaccharide extracted by ultrasound-enzymatic extraction method from head, skin-Esp: the sulfated polysaccharide extracted by enzyme-assisted extraction method from skin, skin-UEsp: The sulfated polysaccharide extracted by ultrasound-enzymatic extraction method from skin.

Generally, the definition of foam is the diffusion of the dispersed phase (usually air) into a continuous phase. The foaming properties of food agents such as polysaccharides and proteins play an important role in food technology [38], [46]. According to previous reports, polysaccharides have a good hydrophilic property which can help to remain in the aqueous sub-phase [27]. Foaming capacity and foam stability of showed a significant difference in all treatments (p < 0.05) (Fig. 4). SPs from head using enzyme-ultrasound method displayed the highest value of foaming capacity and foam stability. It was found that ultrasound pretreatment enhanced the foaming capacity of sulfated polysaccharide from the head and bone, whereas it decreased the foaming capacity of skin samples. According to previous reports, foaming properties were strongly related to molecular weight; lower molecular weights had better foaming properties [31], [40]. As can be seen from the molecular weight results, ultrasound pretreatment could decrease the molecular weight of the sulfated polysaccharides bone and head. This would confirm the obtained results in foam capacity properties. Li et al. [38], who evaluated the effects of ultrasound-assisted extraction on the physicochemical properties of the polysaccharides from *Pholiota nameko* showed that the foaming capacity increased by increasing the ultrasonic treatment time.

#### 4.8.2 Emulsifying properties

**Fig. 5:**
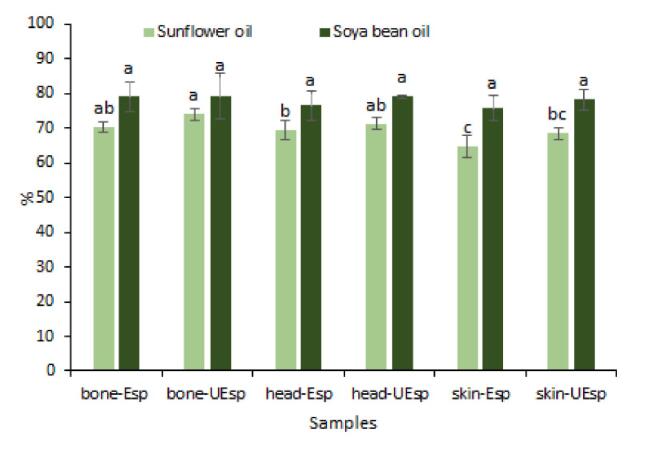
Emulsifying properties of extracted sulfated polysaccharides from Skipjack tuna by-products (skin, bones and head) using ultrasound-enzyme or enzyme extraction method. The results were expressed as mean value ± SD (n = 3). Different letters within the same figure mean statistical difference (p < 0.05). bone-Esp: the sulfated polysaccharide extracted by enzyme-assisted extraction method from bone; bone-UEsp, the sulfated polysaccharide extracted by ultrasound-enzymatic extraction method from bone, head-Esp: the sulfated polysaccharide extracted by enzyme-assisted extraction method from head, head-UEsp: the sulfated polysaccharide extracted by ultrasound-enzymatic extraction method from head, skin-Esp: the sulfated polysaccharide extracted by enzyme-assisted extraction method from skin, skin-UEsp: The sulfated polysaccharide extracted by ultrasound-enzymatic extraction method from skin.

Emulsifying properties is related to the surface activities of polysaccharides. Because of the great surface activities of polysaccharides, they have been drowning attention by the food and cosmetic industry [27], [46]. The emulsifying activities of extracted SPs against sunflower oil and soya bean oil are shown in Fig. 5. Interestingly, extracted SPs from varying parts of fish by-products using different treatments showed excellent emulsifying properties. Ranging from 64.81 % to 79.19 %, the lowest and highest emulsifying capacities were observed in head-UEsp with soya bean oil and skin-Esp with sunflower oil, respectively (p > 0.05). SPs extracted with the aid of ultrasound pretreatment showed slightly highier emulsifying capacity than their counterpart obtained without it but it was not statistically significat. This could be also related to the lower molecular weight of the SPs extacted with ultrasound. Prior studies have mentioned that the emulsifying activity of polysaccharides is associated with some chemical features of polysaccharides such as the acetyl group contents, the neutral sugar composition as well as the protein content of polysaccharide too [19], [27].

### 4.9 Antioxidant activity of SPs

#### 4.9.1 DPPH free radical-scavenging activity

**Fig. 6:**
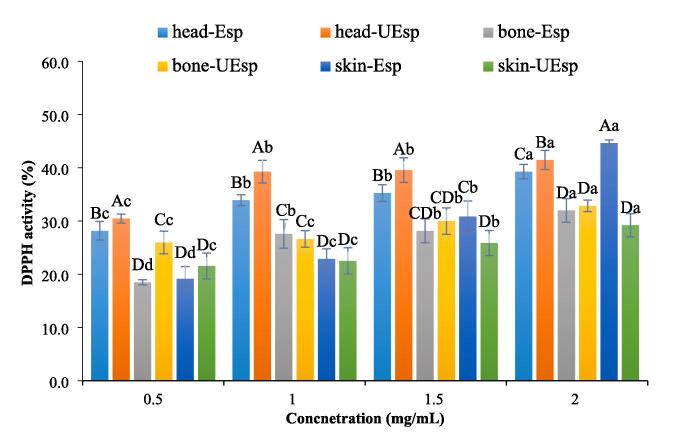
DPPH free radical-scavenging activity of extracted sulfated polysaccharides Skipjack tuna by-products (skin, bones and head) using ultrasound-enzyme or enzyme extraction method. The results were expressed as mean value ± SD (n = 3). Different letters within the same figure mean statistical difference (p < 0.05). The capital letters show the difference between various samples in similar concentrations. The small letters show the difference between various concentrations of the same samples. bone-Esp: the sulfated polysaccharide extracted by enzyme-assisted extraction method from bone; bone-UEsp, the sulfated polysaccharide extracted by ultrasound-enzymatic extraction method from bone, head-Esp: the sulfated polysaccharide extracted by enzyme-assisted extraction method from head, head-UEsp: the sulfated polysaccharide extracted by ultrasound-enzymatic extraction method from head, skin-Esp: the sulfated polysaccharide extracted by enzyme-assisted extraction method from skin, skin-UEsp: The sulfated polysaccharide extracted by ultrasound-enzymatic extraction method from skin.

As shown in Fig. 6, all SPs were capable of donating protons to DPPH free radicals which increased dose-dependently. Also, among all obtained SPs, the highest DPPH free radical-scavenging activity was seen at the concentration of 2 mg/mL as following skin-Esp > head-UEsp > head-Esp > bone-UEsp > bone-Esp > skin-UEsp (p > 0.05). Interestingly, SPs from head and bone treated with ultrasound showed higher DPPH scavenging activity than those extracted with the enzyme treatment by alone at lowe concentrations but this was not seen for SPs from skin. Ultrasound pre-treatment was able to reduce the molecular weight in all treatments but it resulted in an increased sulfate content only in SPs from head and bone which might explain the differences [31], [40]. It has been previously reported that sulfate content, molecular weight as well as the molar ratio of sulfate content to fucose are effective on the antioxidant activity of SPs [30], [48]. Similarly, Li & Wang [15] previously reported that ultrasound-enzyme extraction enhances the antioxidant properties of polysaccharides. SPs extracted from cuttlefish skin and muscle by Jridi et al. [9] showed higher DPPH radical scavenging activity than those extracted in this study.

#### 4.9.2 ABTS antioxidant activity

**Fig. 7:**
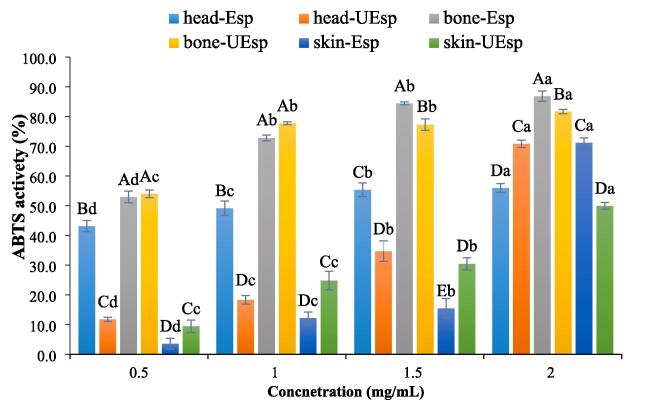
ABTS scavenging activity of extracted sulfated polysaccharides Skipjack tuna by-products (skin, bones and head) using ultrasound-enzyme or enzyme extraction method. The results were expressed as mean value ± SD (n = 3). Different letters within the same figure mean statistical difference (p < 0.05). The capital letters show the difference between various samples in similar concentrations. The small letters show the difference between various concentrations of the same samples. bone-Esp: the sulfated polysaccharide extracted by enzyme-assisted extraction method from bone; bone-UEsp, the sulfated polysaccharide extracted by ultrasound-enzymatic extraction method from bone, head-Esp: the sulfated polysaccharide extracted by enzyme-assisted extraction method from head, head-UEsp: the sulfated polysaccharide extracted by ultrasound-enzymatic extraction method from head, skin-Esp: the sulfated polysaccharide extracted by enzyme-assisted extraction method from skin, skin-UEsp: The sulfated polysaccharide extracted by ultrasound-enzymatic extraction method from skin.

ABTS has been widely applied to estimate the antioxidant capacity of bioactive compounds [19], [49]. As shown in Fig. 7, a wide- range of 3.11 % to 81.66 % of ABTS activity was obtained for the SPs extracted from the tuna by-products. The most powerful ABTS activity was observed in bone-UEsp at 2 mg/mL, while the lowest ABTS activity was measured at 0.5 mg/mL of the skin-Esp. Ultrasonication showed varying effects on the ABTS activity of extracted SPs depending on the constration of the SPs and their source which could be associated with differences in the nature of the used by-product [19]. It has been previously reported that the antioxidant properties of SPs extracted from various sources are extremely affected by their structural characters including molecular weight, conformation and monosaccharides composition [7], [50]. This is in line with the large variation observed in the chemical structure of SPs recovered from the different by-products and with the two methods making explaination of the findings difficult.

#### 4.9.3 Ferrous chelating activity

**Fig. 8:**
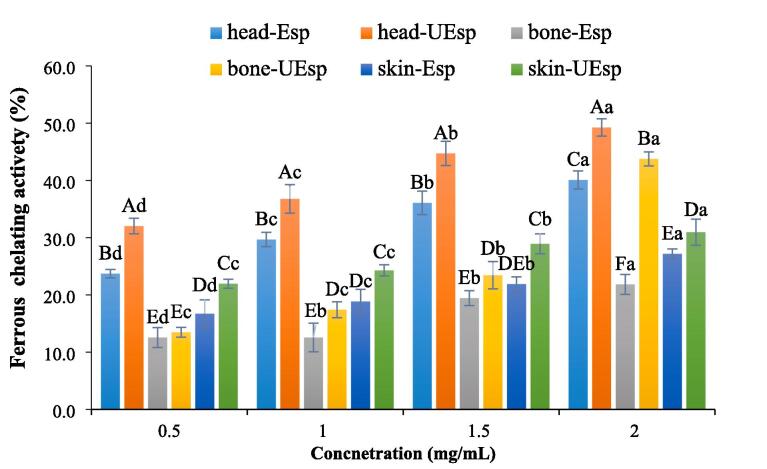
Ferrous chelating activity of extracted sulfated polysaccharides Skipjack tuna by-products (skin, bones and head) using ultrasound-enzyme or enzyme extraction method. The results were expressed as mean value ± SD (n = 3). Different letters within the same figure mean statistical difference (p < 0.05). The capital letters show the difference between various samples in similar concentrations. The small letters show the difference between various concentrations of the same samples. bone-Esp: the sulfated polysaccharide extracted by enzyme-assisted extraction method from bone; bone-UEsp, the sulfated polysaccharide extracted by ultrasound-enzymatic extraction method from bone, head-Esp: the sulfated polysaccharide extracted by enzyme-assisted extraction method from head, head-UEsp: the sulfated polysaccharide extracted by ultrasound-enzymatic extraction method from head, skin-Esp: the sulfated polysaccharide extracted by enzyme-assisted extraction method from skin, skin-UEsp: The sulfated polysaccharide extracted by ultrasound-enzymatic extraction method from skin.

The reaction of Fe^+2^ with hydrogen peroxide (H_2_O_2_) leads to creation of reactive oxygen species (ROS). This reaction happens by Fenton's reaction. The ferrous chelating test is used to assess the antioxidant capacity of bioactive compounds in which the intensity of the purple color of the complex is decreased by adding antioxidant compounds to the ferrozine solution [9], [50]. The results of the ferrous chelating activity of extracted SPs are shown in Fig. 8, The highest ferrous chelating activity was in head-UEsp at 2 mg/mL and the lowest of that was seen for bone-Esp at 0.5 mg/mL [9], [50]. As shown in the results, the ultrasound-enzyme extraction method could enhance the ferrous chelating activity of SPs compared to the enzyme extraction method. Jridi et al. [9] reported that the sulfation of polysaccharides enhanced their chelating capabilities.

### 4.10 Antibacterial activity

Today, there are serious concerns about bacterial infections in developing countries. The increased resistance of bacteria against a wide range of popular antibiotics is also increasing [51]. Here, two Gram-positive (S. aureus and L. monocytogenes) and two Gram-negative (E. coli and S. enterica) bacteria were used to assess the antibacterial activity of SPs, and the results are shown in Table 2. The polysaccharides presented different levels of antibacterial activity against the tested strains. Application of ultrasound treatment to extract SPs from tuna by-products improved the antibacterial activity of the extracted SPs from all by-products against *L. monocytogenes* and *S. aureus* (p < 0.05). It may be due to the highier content of sulphur groups in SPs after ultrasound treatment. All the SPs showed antibacterial activity against *E. coli *(p < 0.05) but it was dose depended. The pretreatment with ultrasound resulted in a slight increase in antibacterial activity of all SPs against *E. coli,* except those from bone. In contrast, ultrasound treatment decreased antibacterial activity of SPs from bone against *S. enterica.* Jridi et al. [5] reported that the antibacterial activity of polysaccharides obtained from Bullet tune bones was higher than 16 mm against S. aureus, M. luteus, B. cereus, K. pneumoniae, and S. enterica, and also a wide range of antibacterial activity was seen against S. aureus, B. cereus, M. luteus, S. enterica, K. pneumoniae, E. coli, Enterobacter for SPs extracted from the skin, bone, and head of Bullet tuna. Li & Shah [52] showed that the introduction of sulfates onto polysaccharides can enhance their capability to disrupt cell walls and cytoplasmic membranes, leading to the dissolution of the proteins and leakage of essential molecules from bacteria resulting in cell death. Besides, there is a more demand to evaluate the influence of polysaccharide chemical composition on the antimicrobial activity, and also bacterial strain type and how bacteria characters can lead to differences in the microbial activity of compounds [51], [53].

**Table 2:** Antibacterial activity of sulfated polysaccharides extracted from Skipjack tuna by-products using enzymatic and ultrasound-enzymatic methods.

by-products Strains	head-Esp		head-UEsp		bone-Esp		bone-UEsp		skin-Esp		skin-UEsp	
	1 mg/mL	2 mg/mL	1 mg/mL	2 mg/mL	1 mg/mL	2 mg/mL	1 mg/mL	2 mg/mL	1 mg/mL	2 mg/mL	1 mg/mL	2 mg/mL
*S. aureus*	0.67 ± 0.58^b^	1.67 ± 0.76^b^	2.67 ± 1.15^b^	2.00 ± 1.50^b^	1.83 ± 0.58^b^	1.67 ± 0.58 ^ab^	2.67 ± 0.29^b^	1.50 ± 0.50 ^ab^	2.17 ± 0.58^b^	1.83 ± 0.29 ^ab^	3.83 ± 0.29 ^a^	2.83 ± 0.76 ^a^
*L. monocytogenes*	non	1.50 ± 0.50^c^	non	1.67 ± 0.68 ^bc^	non	1.67 ± 0.76 ^bc^	1.33 ± 0.29 ^a^	2.00 ± 0.87 ^ab^	1.17 ± 1.26 ^a^	2.67 ± 0.29 ^ab^	1.33 ± 0.29 ^a^	2.83 ± 0.29 ^a^
*E. coli*	1.50 ± 0.50^c^	2.50 ± 0.50 ^ab^	1.67 ± 0.58 ^bc^	2.67 ± 0.58 ^a^	1.33 ± 0.58^c^	1.67 ± 0.58 ^bc^	1.67 ± 0.58 ^bc^	1.50 ± 0.50^c^	2.50 ± 0.50 ^ab^	3.50 ± 0.50 ^a^	2.83 ± 0.29 ^a^	3.50 ± 0.50 ^a^
*S. enterica*	0.33 ± 0.58^b^	0.67 ± 0.58^b^	0.33 ± 0.58^b^	0.67 ± 1.15^b^	0.33 ± 0.58^b^	1.67 ± 0.58 ^ab^	non	1.50 ± 0.50 ^ab^	1.33 ± 0.58 ^a^	1.83 ± 0.29 ^ab^	1.33 ± 0.58 ^a^	2.83 ± 0.76 ^a^

bone-Esp: the sulfated polysaccharide extracted by enzyme-assisted extraction method from bone; bone-UEsp, the sulfated polysaccharide extracted by ultrasound-enzymatic extraction method from bone, head-Esp: the sulfated polysaccharide extracted by enzyme-assisted extraction method from head, head-UEsp: the sulfated polysaccharide extracted by ultrasound-enzymatic extraction method from head, skin-Esp: the sulfated polysaccharide extracted by enzyme-assisted extraction method from skin, skin-UEsp: The sulfated polysaccharide extracted by ultrasound-enzymatic extraction method from skin.

## 5 Conclusions

The effect of ultrasound pretreatment on extraction efficiency of SPs using alcalase from different by-products of Skipjack tuna and their structural, functional and biological activities was evaluated. The highest extraction yield, total sugar, proteins, uronic acid and sulfate were measured in skin-UEsp, bone-Esp, skin-UEsp, skin-UEsp, and bone-UEsp, respectively. FTIR, XRD, and DSC analysis revealed that the extraction process affects on structural properties of SPs from tuna by-products. The extracted SPs showed good foaming properties and great emulsifying capabilities. All extracted SPs showed high antioxidant potential in terms of ABTS, DPPH and ferrous chelating activities where ultrasound treatment enhanced antioxidant activity of the SPs. The SPs exerted significant inhibiting activity against various Gram-positive and Gram-negative bacteria. Ultrasound treatment increased antibacterial activity of the SPs against *L. monocytogenes*. Finally, the results of the present study showed that using ultrasound pretreatment during enzymatic extraction of SPs from tuna by-products can be a promising approach to improve extraction yield SPS but also their bioactivity.

## CRediT authorship contribution statement

**Shahab Naghdi:** Investigation, Methodology, Formal analysis, Writing – original draft. **Masoud Rezaei:** Supervision, Project administration, Conceptualization, Resources, Writing – review & editing. **Mehdi Tabarsa:** Investigation, Writing – review & editing. **Mehdi Abdollahi:** Conceptualization, Writing – review & editing.

## Declaration of Competing Interest

The authors declare that they have no known competing financial interests or personal relationships that could have appeared to influence the work reported in this paper.
